# Bronchiectasis-associated infections and outcomes in a large, geographically diverse electronic health record cohort in the United States

**DOI:** 10.1186/s12890-024-02973-3

**Published:** 2024-04-10

**Authors:** Samantha G Dean, Rebekah A Blakney, Emily E Ricotta, James D Chalmers, Sameer S Kadri, Kenneth N Olivier, D Rebecca Prevots

**Affiliations:** 1 Epidemiology and Population Studies Unit, Division of Intramural Research, National Institute of Allergy and Infectious Diseases, National Institutes of Health , Bethesda, USA; 2 University of Dundee, Ninewells Hospital and Medical School, Dundee, Scotland; 3 Critical Care Medicine Department, Clinical Center, National Institutes of Health, Bethesda, USA; 4 Laboratory of Chronic Airway Infection, Pulmonary Branch, Division of Intramural Research, National Heart, Lung, and Blood Institute, National Institutes of Health, Bethesda, USA; 5 Department of Medicine, Division of Pulmonary Diseases and Critical Care Medicine, University of North Carolina School of Medicine, Chapel Hill, USA

**Keywords:** Bronchiectasis, Respiratory infections, Hospitalization, Exacerbations, Epidemiology

## Abstract

**Background:**

Bronchiectasis is a pulmonary disease characterized by irreversible dilation of the bronchi and recurring respiratory infections. Few studies have described the microbiology and prevalence of infections in large patient populations outside of specialized tertiary care centers.

**Methods:**

We used the Cerner *HealthFacts* Electronic Health Record database to characterize the nature, burden, and frequency of pulmonary infections among persons with bronchiectasis. Chronic infections were defined based on organism-specific guidelines.

**Results:**

We identified 7,749 patients who met our incident bronchiectasis case definition. In this study population, the organisms with the highest rates of isolate prevalence were *Pseudomonas aeruginosa* with 937 (12%) individuals, *Staphylococcus aureus* with 502 (6%), *Mycobacterium avium* complex (MAC) with 336 (4%), and *Aspergillus* sp. with 288 (4%). Among persons with at least one isolate of each respective pathogen, 219 (23%) met criteria for chronic *P. aeruginosa* colonization, 74 (15%) met criteria for *S. aureus* chronic colonization, 101 (30%) met criteria for MAC chronic infection, and 50 (17%) met criteria for *Aspergillus* sp. chronic infection. Of 5,795 persons with at least two years of observation, 1,860 (32%) had a bronchiectasis exacerbation and 3,462 (60%) were hospitalized within two years of bronchiectasis diagnoses. Among patients with chronic respiratory infections, the two-year occurrence of exacerbations was 53% and for hospitalizations was 82%.

**Conclusions:**

Patients with bronchiectasis experiencing chronic respiratory infections have high rates of hospitalization.

## Background

Bronchiectasis is a pulmonary disease defined by the irreversible dilation of the bronchi [1, 2]. Patients typically have a chronic, productive cough and recurring respiratory infections [1], with an associated increased risk of mortality [3]. The current estimated prevalence of bronchiectasis in the United States is up to 213 cases per 100,000 [4] across all age groups, and 700 per 100,000 among adults aged > 65 years [5]. Bronchiectasis has multiple causes including infectious, inflammatory, autoimmune, allergic, and congenital disorders [6, 7]. Recurrent respiratory infections are common and result from impaired mucociliary clearance [8]. These infections trigger inflammation, which in turn worsens underlying damage. Consequently, this vicious cycle leads to increased frequency of exacerbations [1, 8, 9].

Although certain organisms such as *Pseudomonas aeruginosa* and *Staphylococcus aureus* have been associated with exacerbations of bronchiectasis [10], systematic evaluations of bronchiectasis-associated infections in large community and non-tertiary referral populations are lacking. Understanding the etiology and impact of bronchiectasis has implications for effectively treating patients and managing disease [11]. Ongoing cohort studies are expanding our knowledge about the landscape of infections among bronchiectasis patients. In the United States (US), data collected through the US Bronchiectasis Research Registry (BRR) describe infections and treatment among bronchiectasis patients [12]. The European Multicentre Bronchiectasis Audit and Research Collaboration (EMBARC) registry in Europe has recruited more than 20,000 patients as of September 2020 and will provide further insight regarding infections in bronchiectasis patients [13, 14]. However, the US BRR and the EMBARC registry are both based primarily in specialist bronchiectasis clinics and therefore may be biased towards more severe manifestations of the disease. In this study we use a large, nationally distributed Electronic Health Record (EHR) dataset, including microbiological data, to describe bronchiectasis-associated infections and selected outcomes.

## Methods

### Study population

**Fig. 1:**
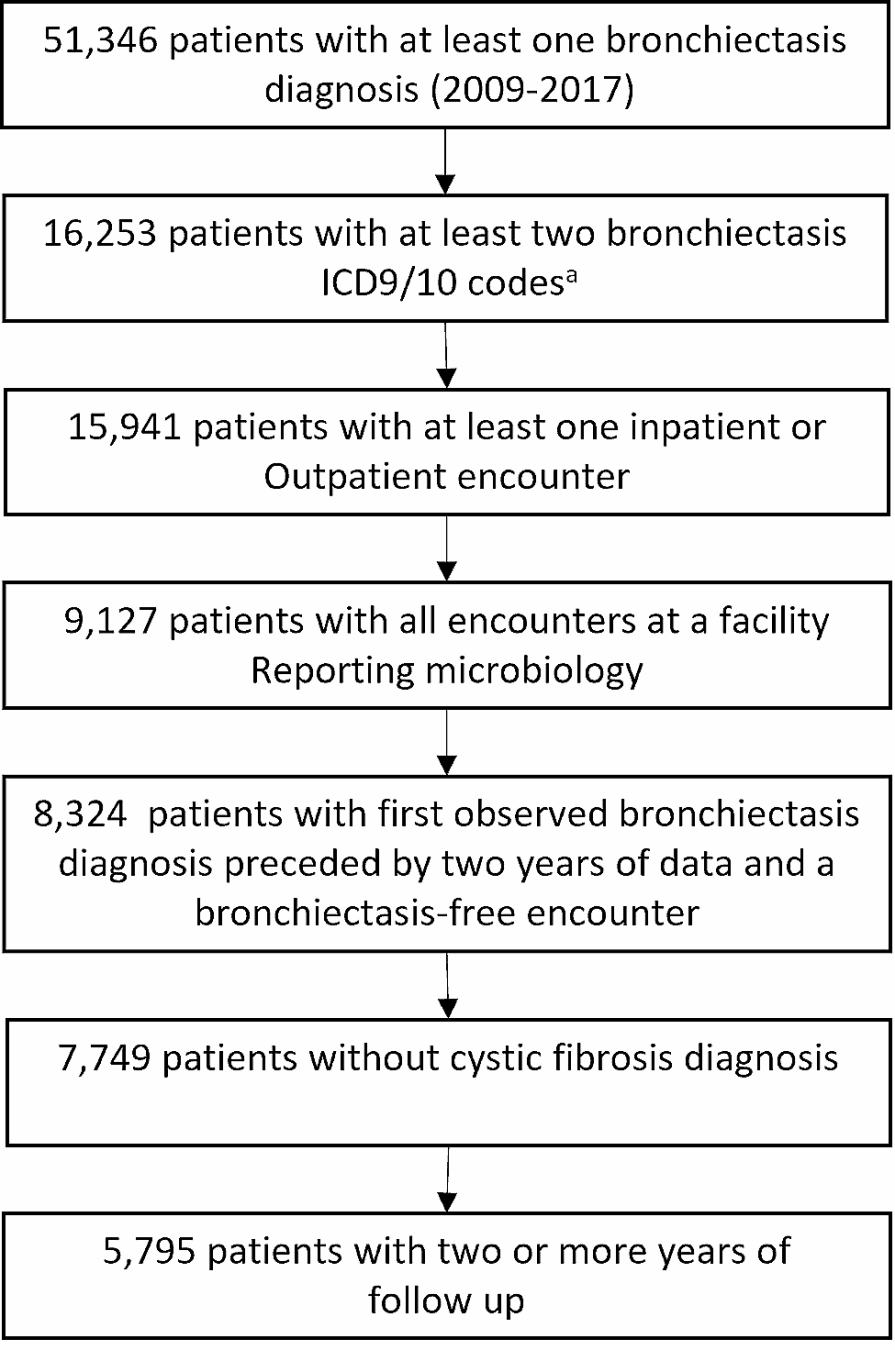
Study population flowchart. ^a^ICD9/10 codes: 494.0, 494.1, 494, 011.50, 011.54, 748.6, 011.51, 011.53, 011.52, 011.55, 011.5, 011.56, J47, J47.9, J47.1, J47.0, Q33.4

Our study population comprised patients in the Cerner *HealthFact*s Electronic Health Record (EHR) database with at least two International Classification of Diseases 9th or 10th revision (ICD9/10) codes for bronchiectasis from 2009 to 2017 (Fig. 1), with no ICD9/10 codes for cystic fibrosis, and where all encounters were in inpatient or outpatient healthcare facilities reporting microbiology data. Facility characteristics are described in Table 1. We considered bronchiectasis cases to be incident if no prior encounters included a bronchiectasis ICD9/10 code for the two years preceding the first bronchiectasis ICD9/10 code (Fig. 1). We included microbiology isolates from only respiratory sites and subset to the most common species isolated, after removing non-pathogenic species and non-speciated results (Table 2). We used text searches for chronic obstructive pulmonary disease (COPD), asthma, and lung cancer to identify ICD codes for these conditions. We defined time under observation as the duration of time between the incident bronchiectasis encounter and the end of the study period.

**Table 1:** Characteristics of inpatient and outpatient facilities in which bronchiectasis patients sought care

Characteristic	No Patients (%)
Bed Size	
< 5	35 (13%)
6–99	84 (32%)
100–199	54 (21%)
200–299	40 (15%)
300–499	30 (12%)
500+	16 (6%)
Teaching Status	
Teaching	151 (58%)
Non-Teaching	82 (32%)
Unknown	27 (10%)
Rural/Urban status^a^	
Rural	55 (21%)
Urban	204 (79%)
Census Region	
Midwest	60 (23%)
Northeast	42 (16%)
South	87 (33%)
West	71 (27%)
Acuity	
Acute	239 (92%)
Non-acute	21 (8%)

^a^One facility missing rural/urban status data

**Table 2:** Definitions of chronic infection and chronic colonization used in analysis

Definition	≥ 2 isolates ≥ 3 months apart within 1 year	≥ 2 isolates on separate days within 2 years	≥ 2 isolates on separate days within 1 year
Organisms	*Pseudomonas aeruginosa* *Staphylococcus aureus* *Haemophilus influenzae* *Streptococcus pneumoniae* *Klebsiella pneumoniae*	*Mycobacterium avium* complex *Mycobacterium abscessus*	*Stenotrophomonas maltophilia* *Aspergillus* sp.

### Data analysis

To estimate the prevalence of organisms associated with bronchiectasis [12], we summed the number of persons in our population with at least one isolate of the selected organisms on or after the date of their first bronchiectasis diagnosis. Whether frequent detection of an organism is considered “infection” or “colonization” varies by organism, thus we also assessed the prevalence using organism-specific definitions of chronic infection or chronic colonization from the literature and expert opinion. For *Mycobacterium avium* complex (MAC) and *M. abscessus*, we defined chronic infection as two or more isolates on separate days within two years of one another [15]. For *Aspergillus* sp [16]. and *Stenotrophomas maltophilia* [17] we defined chronic infection as two or more isolates on separate days within one year of one another. For *Pseudomonas aeruginosa*, we used the definition of chronic colonization established by international consensus and also used in the bronchiectasis severity index (BSI), which counts the number of individuals who had at least two isolates of *P. aeruginosa* three or more months apart within a year [18, 19]. For the remaining species where a more specific definition was not available, we continued to use the EMBARC/BRR chronic colonization definition (Table 2). For calculations of the prevalence of at least one isolate of the specified organism the population denominator was the 7,749 persons who met our case definition for incident bronchiectasis. For purposes of clarity, for the remainder of this paper we will refer to the organism-specific definitions of chronic colonization and chronic infection as “chronic infection.” For calculations of chronic infection prevalence, the population denominator was all persons with at least one isolate of the specified organism.

To describe the impact of chronic infections on clinical outcomes, we evaluated hospitalizations and exacerbations among patients with chronic infection for the most common organisms. For all analyses of chronic infection, we included the 5,795 patients (75% of study population) with at least two years of follow up time after their initial bronchiectasis diagnosis. Hospitalizations were defined as any inpatient encounter. Exacerbations were defined as one or more ICD9/10 codes for bronchiectasis with acute exacerbation or acute respiratory infection, COPD with acute exacerbation, or asthma with acute exacerbation. Codes for asthma and COPD were included to increase the sensitivity of capturing exacerbations. We included a thirty day “window” prior to the incident bronchiectasis diagnosis encounter to include hospitalizations and exacerbations that may have contributed to the identification of bronchiectasis. Rates of hospitalization and exacerbations were calculated for the duration of the study period following the incident bronchiectasis encounter. In addition, because MAC and *P. aeruginosa* are of particular concern among persons with bronchiectasis, we calculated the total time hospitalized using the cumulative time across inpatient encounters. Analysis was completed using R version 3.6.1. We assessed the significance of the difference in proportions of exacerbations and hospitalizations among chronic infection subgroups using two-proportion z-tests with a one-sided alternative and significance assessed at *p* < 0.05. Relative risks of exacerbations and hospitalizations comparing chronic infection vs. no infection were estimated using a univariate negative binomial regression.

## Results

We identified 7,749 persons with incident bronchiectasis, which comprised our study population (Fig. 1). Of these, 5,050 (65%) were women and 5,030 (65%) were aged ≥ 65 years. Concurrent pulmonary disease was common: 3,848 (50%) were diagnosed with COPD, 2,741 (35%) with asthma, and 537 (7%) with lung cancer (Table 3). Overall, persons sought care at 260 unique healthcare facilities, and 65% had all encounters at a single facility within the EHR system during the study period. An additional 24% received care at two facilities.

**Table 3:** Characteristics of study population at incident bronchiectasis encounter

	Category	No Patients (%)
	Total	7,749 (100)
Gender	Female	5,050 (65.2)
	Male	2,699 (34.8)
Race	White	6,154 (79.4)
	African American	723 (9.3)
	Asian	302 (3.9)
	Hispanic	45 (0.6)
	Native American	28 (0.4)
	Pacific Islander	24 (0.3)
Age (Years)	< 65	2,719 (35.1)
	>=65	5,030 (64.9)
Facility teaching status	Non-teaching hospital	3,374 (43.5)
	Teaching hospital	4,053 (52.3)
Facility census region	Midwest	1,827 (23.6)
	Northeast	1,121 (14.5)
	South	2,766 (35.7)
	West	2,035 (26.3)
Concurrent lung conditions^a^	COPD	3,848 (49.7)
	Asthma	2,741 (35.4)
	Lung Cancer	537 (6.9)

^a^ICD9/10 codes: COPD- 496, J44.9, J44.1, J44.0, J44. Asthma- 493.90, 493.92, 493.22, 493.00, 493, 493.20, 493.9, 493.02, 493.91, 493.1, 493.2, E945.7, 493.01, 493.21, 493.11, 493.0, 493.12, 493.82, 493.1, J45.901, J45.909, J45.998, J45.30, J45.31, J45.20, J45.50, J45.991, J45.40, J45.41, J45.51, J45.902, J45.21, T48.6 × 5 A, J45.90, J45, J45.52, J45.42, J45.99, J45.9, J45.32, T48.6 × 6 A, T48.6 × 5D, J45.5, J45.22, J45.4, J45.3. Lung Cancer- 197.0, 162.9, V10.11, 162.5, 162.3, 162.4, 162.2, 235.7, 162.8, V10.12, 162.0, 162, C34.90, Z85.118, C34.11, C34.31, C34.10, C34.92, C78.02, C34.91, C33, C34.00, C34.01, C34.32, C34.12, C34.02, C78.00, C34.2, C34.80, C78.01, C34.82, D38.1, C34.81, C34.30, Z85.11, C34.1, C34.9, Z85.12, C34.8, C34

### Prevalence of infecting pathogens

**Fig. 2:**
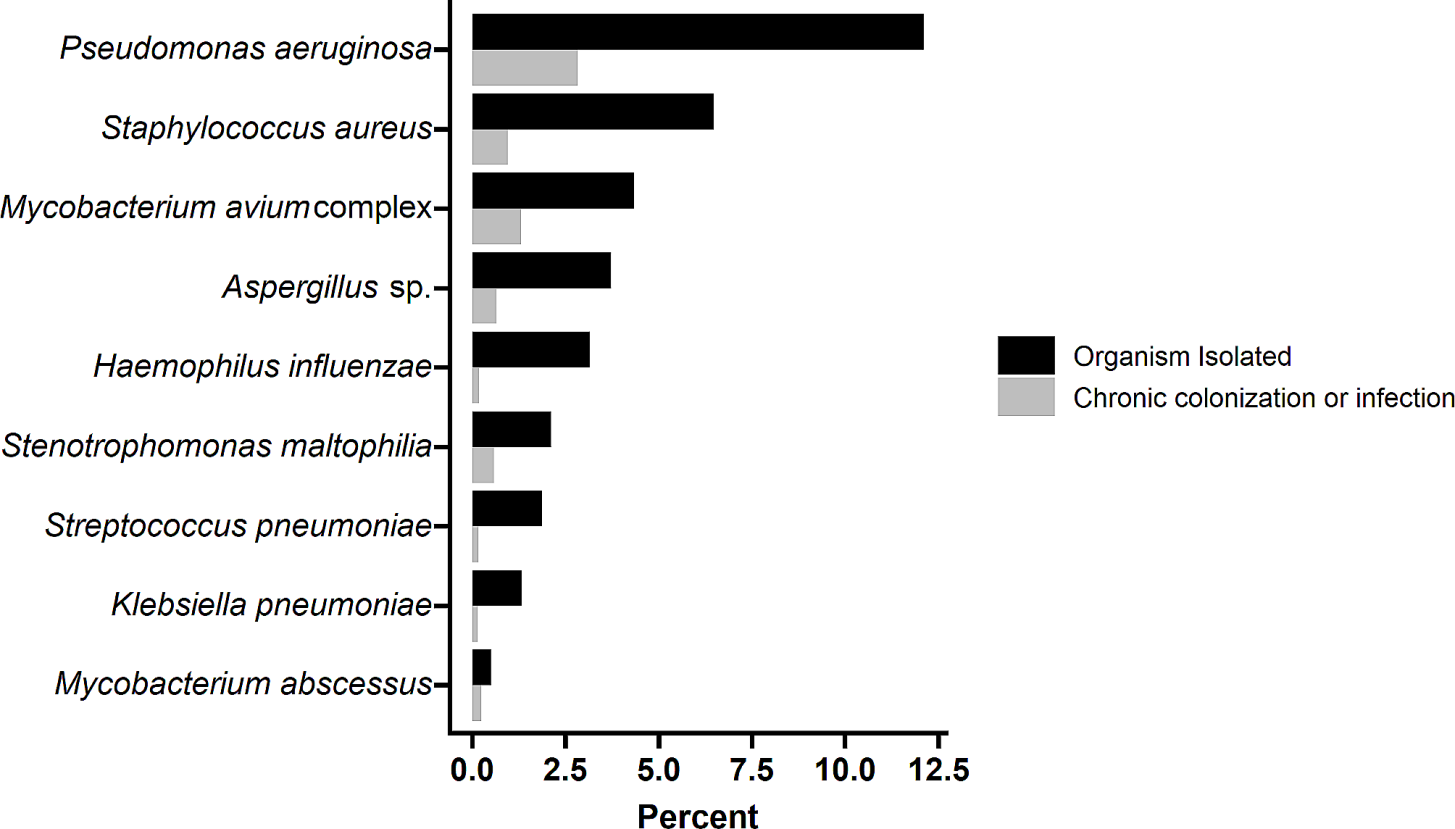
Prevalence of organism isolation and chronic infection for commonly isolated pathogens following incident bronchiectasis diagnosis. See Table 2 for organism-specific definitions of chronic colonization/chronic infection

Of the 7,749 persons in our study population, 4,369 (56%) had at least one pulmonary sample taken for microbiology analysis over an average observation time of 3.6 years per person. Among patients with at least one pulmonary culture, the median number of samples per person was 4 (IQR 2–7): 890 (20%) had one culture, 1597 (37%) had 2–4, 807 (19%) had 5–7, and 1075 (25%) had more than 7. The most commonly identified organisms were *P. aeruginosa* with 937 (12%) individuals, *S. aureus* with 502 (6%), MAC with 336 (4%), and *Aspergillus* sp. with 288 (4%). Of persons with at least one isolate of each respective pathogen, those who met definitions for chronic infection were as follows: 219 (23%) for *P. aeruginosa* colonization, 74 (15%) for *S. aureus*, 101 (30%) for MAC, and 50 (17%) for *Aspergillus* sp. (Fig. 2).

### Infections and clinical outcomes

Of the 5,795 patients with two years of follow up, 1,521 (26%) had an exacerbation within one year and cumulatively, 1,861 (32%) had an exacerbation within two years following the incident bronchiectasis diagnosis (Table 4). Hospitalizations were common, with 3,016 (52%) hospitalized within one year and 3,462 (60%) hospitalized within two cumulative years. A total of 3,954 (68%) patients were hospitalized at any point in the study period after the incident bronchiectasis diagnosis, with a rate of 0.6 hospitalizations per person-year. Among these patients, 920 (23%) had an inpatient encounter including an intensive care unit admission and the 30-day readmission rate was 0.1 per person-year. Inpatient discharge disposition was coded as expired or discharged to hospice for 706 (18%) patients during the study period after the incident bronchiectasis diagnosis.

**Table 4:** Exacerbations and hospitalizations following incident bronchiectasis diagnosis by respiratory co-infection status^a^

	N	Exacerbation					Hospitalization		
		One year (no. %)	Two year (no. %)	Rate per person-year^b^		One year (no. %)	Two year (no. %)	Rate per person-year^b^	
Total	5795	1521 (26)	1861 (32)	0.24		3016 (52)	3462 (60)	0.61	
*Pseudomonas aeruginosa*	139	69 (50)*	89 (64)*	0.86		113 (81)*	121 (87)*	1.42	
*Mycobacterium avium* complex	56	15 (27)*	21 (38)*	0.20		30 (54)*	36 (64)*	0.56	
*Staphylococcus aureus*	44	19 (43)	22 (50)	0.59		34 (77)	36 (82)	1.16	
*Aspergillus* species	35	11 (31)	14 (40)	0.23		30 (86)	31 (89)	0.73	
*Stenotrophomonas. maltophilia*	27	17 (63)	19 (70)	0.75		22 (81)	25 (93)	1.48	
*Mycobacterium abscessus*	12	4 (33)	5 (42)	0.31		9 (75)	9 (75)	0.79	
*Haemophilus influenzae*	8	4 (50)	5 (62)	0.38		4 (50)	5 (62)	0.93	
*Streptococcus pneumoniae*	7	4 (57)	4 (57)	0.73		5 (71)	6 (86)	0.90	
*Klebsiella pneumoniae*	6	3 (50)	3 (50)	0.86		4 (67)	5 (83)	1.39	
Chronic infection with any of above organisms^c^	301	130 (43)	161 (53)	0.59		224 (74)	246 (82)	1.12	
No chronic infection with above organisms^c^	5494	1391 (25)	1700 (31)	0.22		2792 (51)	3216 (59)	0.59	

^a^Exacerbation ICD9/10 codes: 494.1, 493.02, 493.12, 493.22, 493.92; J44.1, J45.21, J45.31, J45.41, J45.51, J45.901, J47.0, J47.1

^b^Time period of 30 days prior to incident bronchiectasis encounter through the end of the study period

^c^Relative Risk of Exacerbations,. chronic infections vs no infections: One year: 1.7 (1.4, 2.0); 2 years; 1.7 (1.5, 2.0); Relative risk of hospitalizations: One year- 1.5 (1.3, 1.7)- Two years- 1.4 (1.2, 1.6)

The 139 patients with chronic *P. aeruginosa* infection experienced significantly more severe clinical outcomes than patients with chronic MAC infection or no chronic infections, with 69 (50%) experiencing exacerbations within one year, compared with 15 (27%) for chronic MAC (*p* < 0.0029) and 89 (64%) experiencing exacerbations within two years compared with 21 (38%) for chronic MAC (*p* < 00064) (Table 4). Patients with chronic *P. aeruginosa* infection also experienced hospitalization more frequently: 113 (81%) patients were hospitalized within one year, compared with 30 (54%) for MAC (*p* = 0.000078) and 121 (87%) were hospitalized within two years, compared with 64% for MAC (*p* < 0.0029). The rates of exacerbations and hospitalizations during the study period were 0.9 and 1.4 per person-year, respectively. The median total duration of hospitalization following incident bronchiectasis diagnosis was longer in the group with *P. aeruginosa* (median 32.6 days, IQR 14.3–61.6) than in the group with MAC (median 10.9 days, IQR 5.3–18) or among patients not chronically infected with any organism of interest (median 11.7 days, IQR 4.8–24.1). Overall, relative to those with no chronic infection,, those with chronic infection with any of the organisms were at a significant 70% increased risk of exacerbations at one and two years of follow-up, and at a significant 40% (one year) to 50% (two years) increased risk of hospitalizations relative to those with no chronic infections (Table 4).

## Discussion

Our study characterized pulmonary infections in a large cohort of patients with bronchiectasis identified through an electronic healthcare record database. Existing estimates of infections and chronic infections among persons with bronchiectasis are based primarily on studies or registries from tertiary care centers with more intensive follow-up, notably the Bronchiectasis Research Registry (BRR) in the US [12] and the European Bronchiectasis Registry (EMBARC). We found that among patients with two years of follow-up, 32% had exacerbations and 60% were hospitalized. Our estimates are similar to a study of bronchiectasis among Medicare patients, which found that 41% of patients had at least one inpatient hospital admission in the 12 months prior to their bronchiectasis diagnosis [5]. Thus, our finding is consistent with another large, population-based sample, particularly given that worsening disease may prompt patients to seek healthcare. Further, a study of a prospective cohort of bronchiectasis with four years of follow up found that 82% of patients with *P. aeruginosa* had a hospitalization related to a severe exacerbation during the study period [18]. This aligns with our finding that 87% of *P. aeruginosa* patients were hospitalized within two years. The high rate of hospitalization following incident bronchiectasis suggests that disease is already somewhat severe by the time of diagnosis. Earlier screening and identification could provide the opportunity for interventions to limit disease progression.

Among incident bronchiectasis cases, the most commonly identified pathogens were *P. aeruginosa*, MAC, *S. aureus, Aspergillus* species. However, the prevalence of infections and chronic infections is likely an underestimate, given that only 56% of patients had any pulmonary sample associated with any encounter over the 9-year study period, with a median of 7 months between diagnosis and first isolate, and only 48% of patients with 2 or more years of observation had more than one isolate. This highlights the need for improved and more systematic evaluation of persons with bronchiectasis, including collection of samples for microbiological analysis.

Eradication therapy for *P. aeruginosa* is recommended to reduce the frequency of poor clinical outcomes such as exacerbation, hospitalization, and mortality, with long-term inhaled antibiotics recommended [20]. Patients with *P. aeruginosa* with frequent exacerbations have worse clinical outcomes, particularly mortality, versus patients with *P. aeruginosa* and without frequent exacerbations. Questions remain regarding how much exacerbations mediate the morbidity and mortality of patients with *P. aeruginosa* and if treatment strategies should vary among patients with *P. aeruginosa* and frequent exacerbation versus those chronically infected but not experiencing frequent exacerbations [21]. The high rate of hospitalization with *P. aeruginosa* may reflect the severity of the infection, the severity of the underlying lung disease or the fact that *P. aeruginosa* is inherently resistant to most oral drugs and therefore intravenous therapy is often required at exacerbation.

Although our dataset represents a large, nationally distributed population, our findings are still subject to limitations inherent in this EHR system. First, because our data are limited to hospitals using the Cerner *HealthFacts* system, we do not have a closed population with regular follow-up. Persons categorized as incident cases of bronchiectasis in our dataset may have an earlier diagnosis in a hospital not represented in this system. The lack of regular follow up also limits our ability to ascertain the sequence of bronchiectasis disease and infection onset. Rather than receiving medical care as disease or infections arise, individuals may have multiple health problems identified at a single, irregular visit. This approach could be an underestimate because we may not have identified persons who had bronchiectasis but were not coded as such, persons who sought care at a facility outside of the Cerner system, or healthcare encounters unrelated to bronchiectasis with no associated bronchiectasis ICD9/10 code. In addition, bronchiectasis ICD9/10 codes have unknown sensitivity and specificity, but are unlikely to identify all true bronchiectasis cases, and could possibly identify more severe cases (given the rarity of this condition relative to other more common pulmonary diagnoses like COPD). Given the small sample size of persons with chronic infections, we could not assess the impact of bacterial coinfections for some pathogens (e.g. Aspergillus). Finally, persons with CF but without an ICD code may have been included in our dataset, with some potential different distribution of organisms; we are unable to evaluate this potential for misclassification in our current dataset. Despite these limitations, the large sample size and the comparability of our findings to other population-based studies speaks to the robust nature of these estimates of infection prevalence. Additionally, our study contributes findings regarding bronchiectasis patients receiving standard clinical care, which is likely more generalizable to all bronchiectasis patients than findings from those patients referred to tertiary care facilities.

## Conclusions

We found a high prevalence of infections and severe outcomes in a nationally distributed population of persons with bronchiectasis, who are likely more representative of all persons with bronchiectasis compared with those enrolled in specialized registries at tertiary care centers. These findings speaks to the need for continued monitoring of lung infections among all persons with bronchiectasis.

## Data Availability

The data that support the findings of this study are available from The Cerner Health Facts, whose dataset was leased by the authors following a Data Use Agreement with the Cerner corporation and as such are not publicly available based on the legal terms of the Data Use Agreement. Interested persons maybe contact the company at the following link: https://www.cerner.com/ap/en/solutions/data-research.
